# Perinatal exposure to methoxychlor enhances adult cognitive responses and hippocampal neurogenesis in mice

**DOI:** 10.3389/fnbeh.2014.00202

**Published:** 2014-06-16

**Authors:** Mariangela Martini, Ludovic Calandreau, Mélanie Jouhanneau, Sakina Mhaouty-Kodja, Matthieu Keller

**Affiliations:** ^1^Physiologie de la Reproduction et des Comportements, UMR 7247 INRA/CNRS/Université Francois RabelaisNouzilly, France; ^2^Physiopathologie des Maladies du Système Nerveux Central, UMR 7224 CNRS/INSERM U 952/Université Pierre et Marie CurieParis, France

**Keywords:** endocrine disruptor, learning and memory, anxiety, methoxychlor, hormones

## Abstract

During perinatal life, sex steroids, such as estradiol, have marked effects on the development and function of the nervous system. Environmental estrogens or xenoestrogens are man-made chemicals, which animal and human population encounter in the environment and which are able to disrupt the functioning of the endocrine system. Scientific interest in the effects of exposure to xenoestrogens has focused more on fertility and reproductive behaviors, while the effects on cognitive behaviors have received less attention. Therefore, the present study explored whether the organochlorine insecticide Methoxychlor (MXC), with known xenoestrogens properties, administered during the perinatal period (from gestational day 11 to postnatal day 8) to pregnant-lactating females, at an environmentally relevant dose (20 µg/kg (body weight)/day), would also affect learning and memory functions depending on the hippocampus of male and female offspring mice in adulthood. When tested in adulthood, MXC perinatal exposure led to an increase in anxiety-like behavior and in short-term spatial working memory in both sexes. Emotional learning was also assessed using a contextual fear paradigm and MXC treated male and female mice showed an enhanced freezing behavior compared to controls. These results were correlated with an increased survival of adult generated cells in the adult hippocampus. In conclusion, our results show that perinatal exposure to an environmentally relevant dose of MXC has an organizational effect on hippocampus-dependent memory and emotional behaviors.

## Introduction

During perinatal life, sex steroids, such as estradiol, have marked effects on the development of the neuroendocrine system and the expression of related behaviors. Indeed, the classical theory of brain sexual differentiation holds that endogenous steroid hormones, during critical periods of development, organize sexual dimorphisms in brain neuronal circuits; these effects are therefore classified as “organizational effect”. Once in adulthood, endogenous steroid hormones then act on these sexually dimorphic neural circuits to give rise to sex differences in behavioral responses (which are classified as “activational effects”).

A variety of man-made chemicals, which animal and human population encounter daily in their environment, are able to disrupt the functioning of the endocrine system. Certain of these artificial environmental contaminants are called “xenoestrogens”, since they mimic the action of naturally produced estrogens (for recent reviews see Frye et al., 2012; Fisher, 2004).

Methoxychlor (MXC) is an organochlorine insecticide that was used to replace Dichlorodiphenyltrichloroethane (DDT) in 1972, due to the adverse effects of DDT on human and wildlife health. MXC was banned in the European Union in 2002^1^ and U.S.A. in 2003,^2^ but it is a persistent chemical and is still found in the environment (Bempah and Donkor, 2011). Moreover, organochlorine pesticides continue to be used for applications such as mosquito and malaria control in developing countries (World Health Organization, 2011) and measurable amounts of MXC and its metabolites can still be found in human tissues, even where the chemical is not used (OSPAR Commission, 2002). In addition, even if the usage of MXC has ceased in the United States and Europe, exposures may still occur via fruits and vegetables imported to U.S. and European markets from other countries that continue to use MXC. Thus, the human health effects of MXC exposure remain an important public health concern.

MXC is an endocrine disrupting chemical (EDC) belonging to the category of xenoestrogens, which acts as an estrogen receptor (ER) α agonist and ERβ and androgen receptor (AR) antagonists (Cummings, 1997). The contamination by MXC can occur by inhalation or dermal contact, but the most effective contamination pathway seems to be through ingestion of contaminated food: MXC is absorbed along the gastrointestinal tract and is then metabolized by the liver following a detoxification pathway (Cummings, 1997). Even though MXC is metabolized by the liver, the estrogenic activity is carried out by its metabolites, which may even have a higher effectiveness than the original compound (Gaido et al., 2000).

Several studies have demonstrated the effects of MXC on reproductive organs in mice: neonatal treatments cause precocious vaginal opening in female mice as well as abnormal ovarian and reproductive tract morphology and premature senescence of the reproductive system in adult females (Eroschenko and Cooke, 1990; Eroschenko et al., 1995). In males, the same treatment inhibits organogenesis of accessory reproductive organs and decreases plasma testosterone levels (Cooke and Eroschenko, 1990; Eroschenko et al., 1995, 1997). Similar effects on reproductive organs have been also observed in male and female rats (for reviews see Chapin et al., 1997; Cummings, 1997; Laws et al., 2000).

MXC effects are also deleterious for the central nervous system, especially during development, leading to disrupted behavioral and sexual differentiation (Palanza et al., 1999, 2001b, 2002; Welshons et al., 1999; Gioiosa et al., 2007). Indeed, oral administration of MXC during the perinatal sensitive period causes organizational estrogen-like changes in behavior in female (increase of motor activity; Gray et al., 1988) and male rats (disruption of sexual behavior; Gray et al., 1989). In mice, embryonic exposure to MXC causes changes in reflex development, delayed onset of male intrasex aggression and increased rate of urine marking (Palanza et al., 1999).

While scientific interest in the effects of exposure to xenoestrogens has focused more on fertility and reproductive behavior effects, the effects on cognitive function have received less attention. This matter of fact seems to be surprising because, besides their importance for reproduction, estrogens play also a critical role in many higher cognitive abilities in vertebrates (McEwen, 2002; Rapp et al., 2003; Scharfman and Maclusky, 2005). For example, hippocampal memory dependent functions, such as spatial memory, are sensitive to estrogen in a variety of species including humans (Lephart et al., 2004).

The present study explored, for the first time, whether MXC administered during the perinatal period to male and female mice, at concentration that influenced other aspects of development (Palanza et al., 1999, 2001b, 2002; Gioiosa et al., 2007), would also affect learning and memory functions, especially those depending on the hippocampus. In addition, we wondered whether perinatal exposure to this EDC can also influence hippocampal neurogenesis, a form of plasticity based on persistent formation of adult-born neurons in the hippocampal dentate gyrus (DG). This is of particular importance as hippocampal neurogenesis participates in hippocampal-dependent memory (Koehl and Abrous, 2011).

## Materials and methods

### Maternal treatment and procedure

Swiss CD1 mice were originally purchased from Janvier Breeding Center (Le Genest Saint-Isle, France) and were bred in our animal facility. Adult (2–3 months old) virgin females were time-mated by being placed into the cage of a stud male for one night, beginning at 18:00 h (at the end of the light phase of the 12-h light/dark cycle). Mating was verified by the vaginal plug presence (gestational day 0). After mating pregnant females were housed three per cage (45 × 25 × 15 cm^3^) until the treatment started.

MXC was administered at a dose of 20 µg/kg (body weight)/day, which has been shown to affect behavioral development in previous experiments (Palanza et al., 2001b, 2002; Gioiosa et al., 2007). It is well below the 50 mg/kg/day lowest observed adverse effect level (LOAEL) and the 5 mg/kg predicted no observed adverse effect level (NOAEL; World Health Organization, 1996). This environmentally relevant dose is within the human exposure range and is considered as not teratogenic (see Brotons et al., 1995; Olea and Olea-Serrano, 1996; vom Saal and Hughes, 2005; Welshons et al., 2006). From gestational day 6, pregnant dams were daily trained to drink a small volume (0.1 ml) of sesame oil from a modified micropipette (i.e., with a larger hole). This training ensured that the treatment procedure was not stressful (Palanza et al., 2002). On gestational day 11, females were individually housed and randomly assigned to one of the following treatment groups: sesame oil (control; *N* = 7) or MXC at 20 µg/kg/day (Sigma Chemical; *N* = 7) dissolved in sesame oil. Each female was fed 0.1 ml/50 g body weight/day of sesame oil with or without MXC 4 h after light onset and from gestational day 11 to postnatal day (PND) 8. During this critical period for brain development in mice and rats, estrogens organize permanently and irreversibly specific brain circuitries (Arnold and Breedlove, 1985; Bigsby et al., 1999).

After dams gave birth, litters were sexed, culled to 10 pups (5 ± 1 males and 5 ± 1 females) and returned to their mothers in the first 12 h of postnatal life. The offspring were weaned on PND 21 and group-housed with the same-sex littermates (5 mice/cage) during the whole experiment in 45 × 25 × 15 cm^3^ cages at 22 ± 1°C, with a 12-h reversed dark/light cycle (lights off at 10:00 h). Water and pellet food (Safe, Augy, France) were available *ad libitum*. All animal studies were performed in accordance with the French and European legal requirements (Decree 2010/63/UE).

### Experimental subjects

All behavioral tests were performed on one cohort of male and female control (OIL) and treated (MXC) mice (*N* = 12–15 per treatment group) between 10:00 h and 18:00 h. The sequence of the tests was performed as follows: 1/elevated plus-maze (EPM), 2/openfield, 3/forced swim test (FST), 4/spontaneous alternation in a Y-maze and 5/the most aversive test, contextual fear conditioning. To allow a full recovery between tests, especially for those involving an aversive component, tests were spaced by a 1 week interval. A different cohort of animals was used for 5-Bromo-2-Deoxyuridine (BrdU) injections (*N* = 6 per treatment group) so that the level of BrdU expression was not influenced by behavioral tests. To avoid any litter effect, no more than two pups originating from the same litter were included in the same experimental group for behavioral experiments; these two animals were tested in all experimental paradigms. For the BrdU experiment, no more than one pup originating from the same litter was included in the same experimental group. All tests were performed in a room adjacent to the room where animals were housed.

As the hormonal status may influence female responses (Marcondes et al., 2002; Simpson and Kelly, 2012), the phase of the estrous cycle was determined by vaginal smear cytology for each female before the behavioral tests. Cell type composition of the vaginal smears was determined (as described in McLean et al., 2012) and only females at the diestrus stage, thus showing low and stable levels of ovarian hormones (Butcher et al., 1974), were used.

### Measurement of anxiolytic-like behavior in the elevated plus-maze (EPM)

The elevated plus-maze (EPM) consisted of a black plastic apparatus with four arms (16 × 5 cm) set in a cross from a neutral central square (5 × 5 cm). Two opposite arms were delimited by vertical walls (closed arms) whereas the two other opposite arms had unprotected edges (open arms). The maze was elevated 40 cm above the ground. At the beginning of the 5-min test session, each mouse was placed in the central neutral area, facing one of the open arms. The total time spent in the open arms was then scored. Time spent in the open arms was recorded when the mouse moved two forepaws and head into the arm. Results were expressed as the percentage of time spent in the open arms.

At 2 months of age, all subjects were tested for their behavior in the EPM as previously described (Keller et al., 2010; Martini et al., 2014). Control and MXC-treated mice were brought into the test room at least 1 h before the onset of behavioral testing and remained in the same room throughout the test. Mice were tested individually, during the dark phase of the dark-light cycle in a random order. The maze was cleaned with 70% ethanol to eliminate odors after each test.

### Measurement of anxiety and locomotor activity in the open-field test (OFT)

Each animal was placed in an open field apparatus consisting of a rectangular area (40 × 40 × 30 cm^3^ (Martini and Valverde, 2012)). A total of 16 squares (10 × 10 cm) were drawn with black lines on the white floor dividing the field into central and peripheral areas. To start the experiment, each mouse was placed in the central area of the field. The parameters measured during a 5-min observation period were the time spent in the central area of the field, as indicator of anxiety-like behavior, and the total number of crossed squares, as indicator of locomotor activity.

### Measurement of depressive-like behavior in the forced swim test (FST)

Mice were placed into an opaque cylinder tank (24 cm diameter, 53 cm height) filled to a depth of 30 cm with water maintained at 25°C to 27°C. The water was changed after each animal, and the tank was thoroughly cleaned. Mice were placed for 6 min in the cylinder and scored for time spent actively swimming vs. floating (no leg or tail movement contributing to forward movement). The amount of time the mice spent floating during the last 4 min of the trial was recorded. An increase in floating is interpreted as an increase in depressive-like behavior (Porsolt et al., 2001).

### Assessment of working spatial memory through spontaneous alternation in the Y-maze

Spontaneous alternation was assessed in a Plexiglas Y-maze apparatus, elevated 80 cm from the floor and located in the middle of a room containing a variety of extra-maze cues. The walls of the maze were 10 cm high; each arm was 50 cm long and 12.5 cm wide.

The test consisted of two visits in the apparatus separated by 90 s or 24 h, to assess short- and long-term memory respectively (Sanderson et al., 2009). Briefly, during the first visit (sample phase), mice were placed at the end of a chosen arm (start arm) and allowed to explore two arms of the maze (the “start” arm and one of the two other arms called the “other” arm) for 5 min with the third arm closed (“novel” arm). Then, mice were removed from the maze and placed in a waiting cage (similar to the home cage) during a 90-s period to assess short-term memory or to their home cage during a 24-h period to evaluate long-term memory. During the second visit (test phase), the mice were placed at the end of the start arm and allowed to explore freely all three arms of the maze for 2 min (the start, the other and the novel arm). Allocation of arms (other and novel) was counterbalanced within each experimental group. During the sample and the test phases, the time spent in each accessible arm was measured. The maze was cleaned with 70% ethanol between trials.

### Contextual fear conditioning test

Fear conditioning was conducted in an operant chamber installed in a ventilated, sound insulated chest (48 × 36 × 40 cm). The chamber (26 × 18 × 23 cm) was a Plexiglas box equipped with a grid floor made of stainless steel rods (2 mm in diameter), spaced at intervals of 6 mm center-to-center, through which scrambled electric shock could be delivered (Imetronic, Talence, France). Mice were held outside the experimental room in their home cages prior to testing and transported to the conditioning apparatus individually in standard mouse cages. Chamber was cleaned with 70% ethanol between each mouse.

The test consisted of two phases: (a) conditioning and (b) test of conditioned freezing to the same context. Conditioning was conducted on day 1 and consisted of two unsignaled deliveries of a 1-s, 0.6 mA footshocks. The first shock was delivered 3 min after the mice were placed into the chamber. Successive shock was delivered 3 min later and mice were removed 2 min after this second shock. On day 2, all mice were returned in the chamber. They were placed in the chamber for 8 min, and freezing levels were recorded in the absence of any further stimulus.

### 5-Bromo-2-Deoxyuridine (BrdU) injections and tissue preparation

To assess levels of cell survival in the hippocampal DG, adult mice received four i.p. injections of BrdU (50 mg/kg in 0.9% saline; Sigma-Aldrich, Saint-Quentin Fallavier, France), with 2 h intervals (between 11:00 and 17:00 h) and were killed 21 days later. At the end of the experiment, animals were anesthetized and transcardially perfused with 1% sodium nitrite in phosphate buffer saline, followed immediately by 4% cold paraformaldehyde solution in 0.1 M phosphate buffer (pH 7.4). Brains were removed and post-fixed in 4% paraformaldehyde for 2 h and they were then placed overnight in a 30% sucrose solution in PBS, frozen in liquid isopentane at −35°C and stored in a deep freezer at −80°C until sectioning.

Brains were serially cut in the coronal planes at 30 µm thickness with a cryostat. The plane of sectioning was oriented to match the drawings corresponding to the coronal sections of the mouse brain atlas (Franklin and Paxinos, 1997). Serial sections were collected in a cryoprotectant solution at −20°C (Watson et al., 1986). Every fourth section (a section every 120 µm) was processed for BrdU immunohistochemistry and for BrdU/NeuN double immunofluorescence labeling.

### BrdU immunohistochemistry

To reveal BrdU positive cells, a peroxidase single-immunolabeling was used. Sections were treated with TBS 0.025 M, pH 7.4—Triton 0.3%—Azide 0.1%—BSA 1% (TBSTA-BSA) for 1 h to increase permeability of plasma membrane. After one rinse in TBS, sections were treated with 2N HCl in TBS for 25 min at 37°C in order to denaturate the DNA. After three rinses in TBS, sections were incubated overnight in primary rat anti-BrdU (1:300; AbCys AbC117-7513, Paris, France) in TBSTA-BSA. The next day, sections were rinsed three times in TBS, and were incubated for 1:30 h with a goat anti-rat peroxidase (1:300; Jackson 112-036-003, USA) in TBS 0.025 M, pH 7.4—Saponine 0.3%—BSA 1% (TBS-Saponine-BSA). Finally, after two rinses in TBS and two rinses in Tris-HCl 0.05 M, pH 7.6, sections were reacted for peroxidase detection in a solution of 3,3′-Diaminobenzidine tetrahydrochloride (0.15 mg/ml; Sigma) containing 0.001% H_2_O_2_ and 0.46% nickel ammonium sulphate (DAB-Ni- H_2_O_2_ revelation).

### Double immunofluorescence labeling BrdU/NeuN

To characterize the phenotype of BrdU positive cells in the DG, we used NeuN, a marker for postmitotic neurons. A double immunofluorescence labeling was performed against BrdU and NeuN. Coronal sections of the hippocampus were treated with TBS 0.025 M, pH 7.4—Triton 0.3%–Azide 0.1%–BSA 0.1% (TBSTA-BSA) for 1 h to increase permeability of plasma membrane. After one rinse in TBS, sections were treated with 2N HCl in TBS for 25 min at 37°C in order to denaturate the DNA. After three rinses in TBS, sections were incubated overnight in primary antibodies (BrdU, 1:300, AbCys AbC117-7513, Paris, France; NeuN, 1:1000, Chemicon MAB377, clone A60, Millipore, St. Quentin-en-Yvelines, France) in TBSTA-BSA. The next day, sections were rinsed three times in TBS, and were incubated for 1:30 h with CY3Donkey anti-rat (1:300, Jackson ImmunoResearch 712-165-153, United Kingdom), and 488 donkey anti-mouse (1:300, AlexaFluor A11029, Molecular Probes, Eugene, Oregon, USA) in TBS 0.025 M, pH 7.4—Saponine 0.3%–BSA 1% (TBS-Saponine-BSA). After four rinses in TBS, sections were immersed in a Hoechst solution for 2 min (Hoechst 33258, 2 µg/ml in water, Invitrogen, USA), rinsed twice in water, dried and then cover-slipped under fluoromount-G (Southern-Biotech, Birmingham, AL, USA) and stored at +4°C in the dark.

### Quantification

The number of BrdU positive cells was assessed by counting peroxidase/DAB-stained coronal sections of the DG with a light microscope on a magnification of 20x and cell count analysis software (computerized image analysis: Mercator, Explora Nova, La Rochelle, France). The counter was blind to the experimental group. Six coronal sections were selected through the dorsal hippocampus. Areas of the granule cell layer (GCL) and the subgranular zone (SGZ) of the DG were delimited and measured with an 10x objective. The density of BrdU positive cells was then calculated by dividing the numbers of BrdU positive cells by the layer area.

To determine the proportion of new cells, which expressed BrdU and NeuN, a confocal laser-scanning microscope (LSM 700, Zeiss, Germany) was used. The corresponding six coronal sections of the BrdU positive cells counting were selected through the dorsal hippocampus. Each BrdU positive cell was analyzed in its entire *z*-axis, with 0.5 µm step intervals, using an 40x oil-immersion objective to identify double immunolabeling.

### Statistical analysis

All the analyses were performed using the software Statistica 6.0 (StatSoft Inc., Tulsa, OK, USA). Differences were considered significant when *p* < 0.05. Data are represented as mean ± SEM. Effects of MXC in male and female mice were compared using two-way analyses of variance (ANOVA), with sex and perinatal exposure as independent factors. Significant effects revealed by ANOVA were further explored using the *post-hoc* Fisher’s LSD test. The potential litter effect was statistically evaluated using a one-way ANOVA, with litter as independent factor. However, as no significant litter effect was detected, we did not present these results in the following section.

## Results

### Elevated plus-maze (EPM)

**Figure 1:**
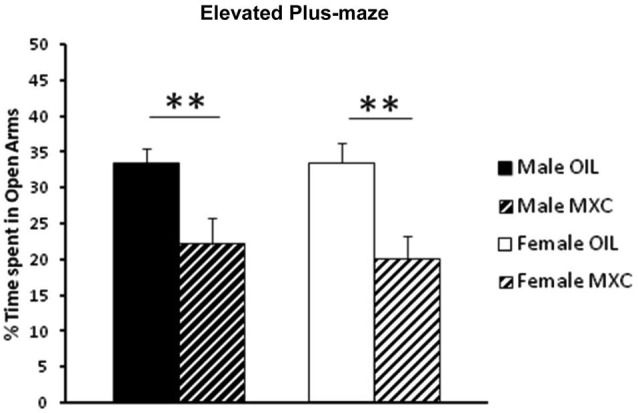
**Percent of open-arm time in the EPM**. Perinatal exposure to MXC in both male and female mice revealed a significant decrease in the percentage of time spent in the open arms compared to control groups. Data are expressed as mean ± SEM. ** *p* < 0.01.

A two-way ANOVA (with sex and perinatal exposure as independent factors) on the percentage of time spent in the open arm revealed a significant effect of the treatment (*F*_(1,46)_ = 17.690, *p* < 0.001), but no effect of sex (*F*_(1,46)_ = 0.107, *p* = 0.745) or interaction between sex and treatment (*F*_(1,46)_ = 0.126, *p* = 0.724). In fact, both male and female mice perinatally exposed to MXC exhibited a significant increase in anxiety-like behavior evaluated in the EPM compared to control animals, since the percentage of time spent in the open arms was lower compared to control groups (Figure 1).

### Open field test (OFT)

**Figure 2:**
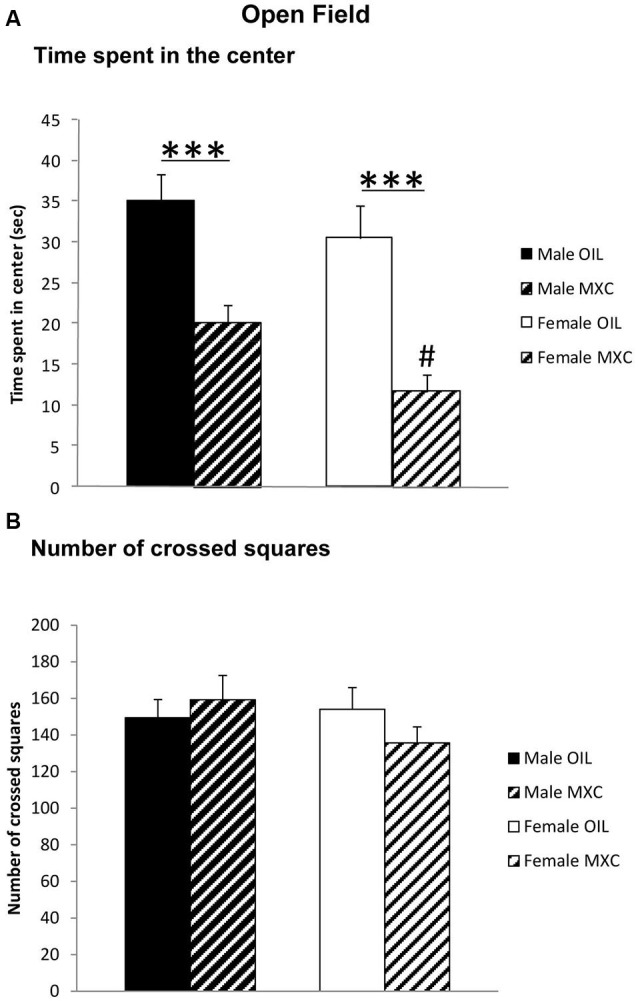
**(A)** Time spent in the central area and (B) total number of crossed squares in an open field. Perinatal MXC exposure induced a decrease in the time spent in the central area in female compared to male mice and a significantly decrease in MXC treated mice compared to control mice. No differences of the total number of crossed squares were detected, as indicator of locomotor activity. Data are expressed as mean ± SEM. *** *p* < 0.001; # *p* < 0.05 males vs. females.

Different responses were evaluated in the open-field test (OFT): the time spent in the central area of the field, as indicator of anxiety-like behavior, and the total number of crossed squares, as indicator of locomotor activity. The time spent in the central area was significantly lower in females by comparison with males (sex: *F*_(1,56)_ = 4.604, *p* = 0.036), and significantly decreased in MXC-treated mice compared to controls (treatment: *F*_(1,56)_ = 31.774, *p* < 0.001; sex*treatment: *F*_(1,56)_ = 0.402, *p* = 0.529) (Figure 2A).

Two-way ANOVA revealed no effects of sex or treatment on locomotor activity (total number of crossed squares) (sex: *F*_(1,56)_ = 0.735, *p* = 0.395; treatment: *F*_(1,56)_ = 0.151, *p* = 0.699; sex*treatment: *F*_(1,56)_ = 1.604, *p* = 0.211) (Figure 2B).

### Forced swim test (FST)

**Figure 3:**
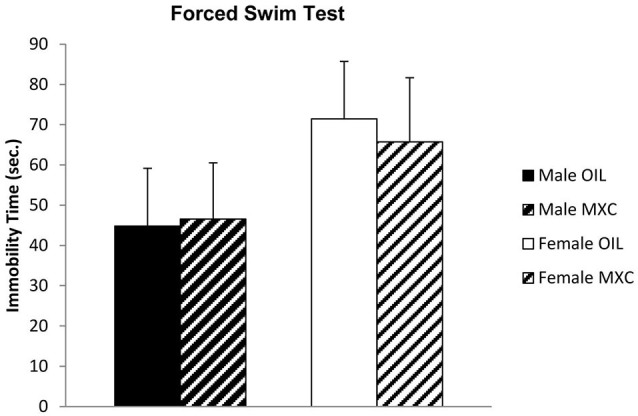
**Time spent floating, in seconds, in the FST.** Male and female mice perinatally exposed to MXC exhibited no effects in the depressive-like profile evaluated in the FST. Data are expressed as mean ± SEM.

Male and female mice exposed to MXC during the perinatal period exhibited no difference in the depressive-like profile evaluated in the FST. Two-way ANOVA indicated no effect of sex (*F*_(1,44)_ = 2.441, *p* = 0.125), treatment (*F*_(1,44)_ = 0.018, *p* = 0.894), and no interaction between these two factors (*F*_(1,44)_ = 0.064, *p* = 0.801; Figure 3) on the time the animal spent floating during the recorded period.

### Spontaneous alternation in the Y-maze

**Figure 4:**
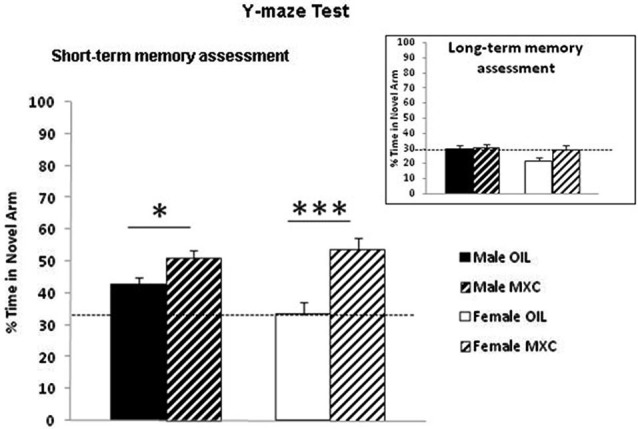
**Enhanced short-term working spatial memory through spontaneous alternation in the Y-maze in MXC-treated mice.** The percentage of time spent in the novel arm relative to the total duration of visits in the three arms during the test phase revealed an effect of the perinatal MXC treatment. Data are expressed as mean ± SEM. * *p* < 0.05; *** *p* < 0.001. In the right corner: assessment of long-term memory after 24 h revealed no significant effects of MXC treatment.

Regarding short-term memory assessment, two-way analysis of the percentage of time spent in the novel arm [(time spent in the novel arm divided by three arms) × 100], revealed an effect of the treatment (*F*_(1,56)_ = 20.282, *p* < 0.001) and an almost significant interaction sex*treatment (*F*_(1,56)_ = 3.706, *p* = 0.059), but no effects of sex (*F*_(1,56)_ = 1.013, *p* = 0.318) during the test phase (Figure 4).

This effect was specific of short-term memory, because when long-term memory was assessed after 24 h, two-way ANOVA of the percentage of time spent in the novel arm, revealed no significant effect of the treatment, of sex and no interaction sex*treatment (*F*_(1,56)_ = 3.242, *p* = 0.077; *F*_(1,56)_ = 1.981, *p* = 0.165; *F*_(1,56)_=1.313, *p* = 0.257 respectively) during the test phase (Figure 4).

### Contextual fear conditioning test

**Figure 5:**
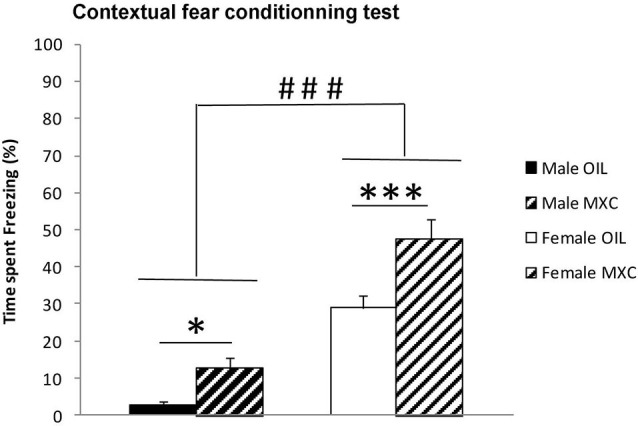
**Freezing behavior expressed as percentage of time spent freezing during the recorded period in contextual fear conditioning test.** The percentage of time that the animal spent freezing revealed a significant effect of sex, with the females spending a greater percentage of time freezing than males. Percentage of contextual freezing was also significantly higher in MXC-treated mice compared to control. Data are expressed as mean ± SEM.* *p* < 0.05; *** *p* < 0.001; ### *p* < 0.001 in Females vs. Males.

Two-way ANOVA calculated for the percentage of time the animal spent freezing during the recorded period revealed a significant effect of sex (*F*_(1,56)_ = 80.880, *p* < 0.001) with the females spending a greater percentage of time freezing than males. The percentage of contextual freezing was also significantly higher in MXC treated mice compared to control mice (significant effect of the treatment: *F*_(1,56)_ = 18.289, *p* < 0.001; sex*treatment: *F*_(1,56)_ = 1.576, *p* = 0.215). Subsequent *post-hoc* Fisher’s LSD analysis revealed an increase in the time spent freezing in MXC treated mice (*p* = 0.04 and *p* < 0.001 in male and female mice respectively) (Figure 5).

### Hippocampal neurogenesis

**Figure 6:**
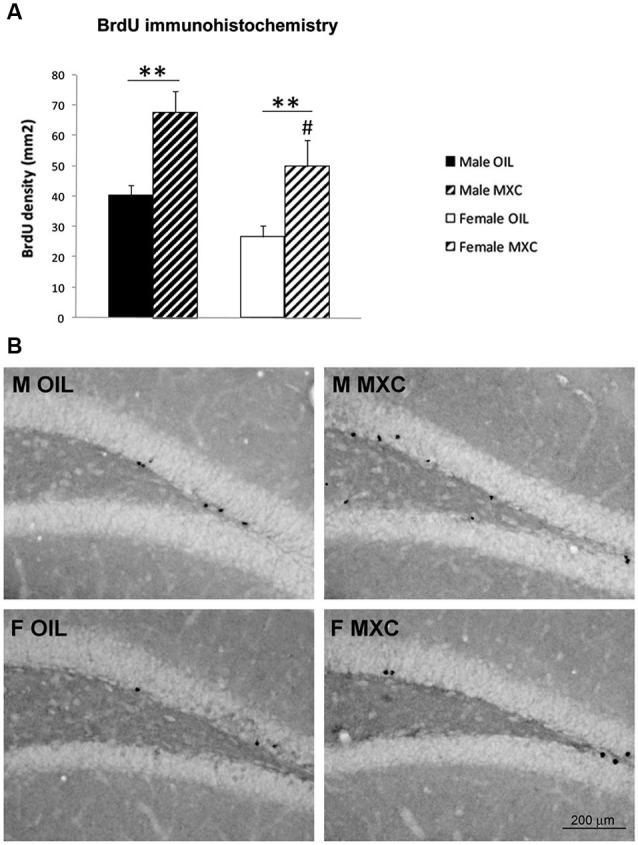
**Perinatal MXC exposure increased BrdU+ cells in the dentate gyrus of the hippocampus. (A)** The density of BrdU+ cells increased in both sexes in MXC-treated mice. Density of cells survival was higher in male compared to female mice. Data are expressed as mean ± SEM. ** *p* < 0.01; # *p* < 0.05 in Males vs. Females. **(B)** Representative BrdU staining in the dentate gyrus (DG) of the hippocampus for each group.

The perinatal MXC treatment led to a significant increase in cells survival in the DG of the hippocampus in both sexes compared to corresponding controls. In addition, density of cells survival was higher in males compared to females. Indeed, two–way ANOVA (sex and treatment as independent factors) revealed a significant effect of sex and treatment (*F*_(1,20)_ = 6.742, *p* = 0.017 and *F*_(1,20)_ = 17.698, *p* < 0.001 respectively) but no interaction between both factors (*F*_(1,20)_ = 0.129, *p* = 0.723; Figures 6A–B).

**Figure 7:**
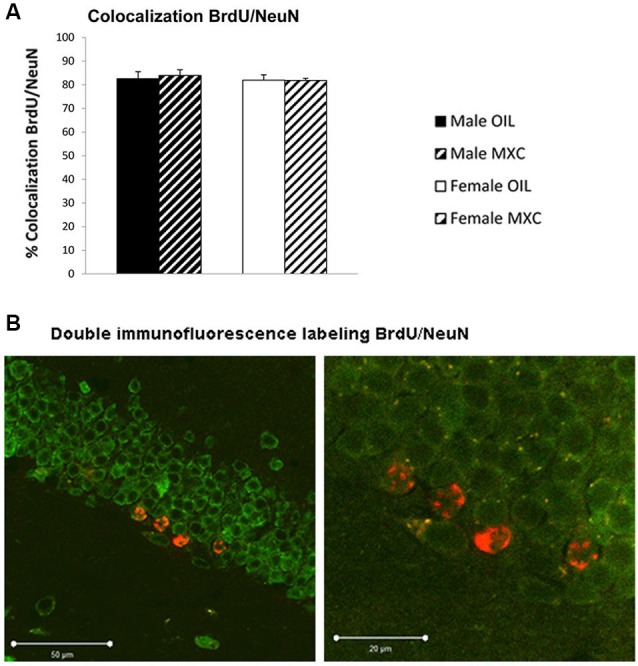
**Number and phenotyping of BrdU+ cells in the dentate gyrus of the hippocampus. (A)** The percentage of BrdU+ cells that expressd NeuN did not significantly differ according groups. Data are expressed as mean ± SEM. **(B)** Representative confocal BrdU(red)/NeuN(green) double-labeled images in the DG of the hippocampus at low (left panel) and high (right panel) magnification.

The percentage of BrdU-positive cells expressing NeuN in the DG of hippocampus (83% ± 2.1) did not significantly differ between groups. Two-way ANOVA revealed no effects of sex (*F*_(1,20)_ = 0.300, *p* = 0.590), treatment (*F*_(1,20)_ = 0.028, *p* = 0.870) and no interaction between these two factors (*F*_(1,20)_ = 0.064, *p* = 0.803; Figures 7A–B).

## Discussion

While the effects of exposure to xenoestrogens have been largely documented on fertility and reproductive behaviors, the effects on cognitive function have received less attention. Thus, an important aspect of this study is that our data extend the adverse effects of MXC to non-reproductive behaviors at an environmentally relevant dose within the human exposure range. The overall result of our study is that maternal exposure to a very low dose of MXC during fetal and neonatal periods had an enduring effect on hippocampus-dependent memory and plasticity as well as on emotional behaviors in adulthood.

We first assessed explorative and emotional behavior in EPM and OFT. We found no difference in control male and female groups in both experimental paradigms, but both sexes perinatally exposed to MXC showed an increase in anxiety-like behaviors: MXC exposed mice spent less time than controls in the open arms of the EPM, and central area of the OFT. Since the number of crossed squares in the OFT did not vary between control and MXC exposed mice, the differences encountered are not related to a possible locomotor impairment induced by MXC.

We have explored whether this increased anxiety effect of MXC could also lead to depressive-like behavior. No effect of MXC treatment was detectable on the time the mice spent floating in the FST. Thus we concluded that perinatal MXC exposure specifically increased anxiety without inducing any depressive-like phenotype.

Our results in the EPM are in agreement with data obtained in the OFT, but also with previous behavioral tests in female mice (Palanza et al., 2002; Gioiosa et al., 2007) showing that MXC exposure lead to an anxiogenic effect, with a decrease in the time spent in the open arms of the EPM and a lower exploration of the OFT. By contrast, the effects observed in male mice in our experiment are not in accordance with those reported in previous studies. Indeed, sex differences were observed in control mice in both EPM and OFT, with unexposed males being more anxious and less prone to explore a novel environment when compared with control females (Gioiosa et al., 2007). In addition, perinatal exposure to MXC, at the same dose than that used in our study, did not induce any effect in males (Gioiosa et al., 2007). Although the reasons for this discrepancy are not completely clear, it must be noticed that sex differences in anxiety-like behavior, for example when measured in the OFT, may be not always observed in CD-1 mice, the strain used in the present study (Palanza et al., 2001a). In addition, in other experiments, using perinatal exposure to daidzein, a phytoestrogen compound with higher estrogenic activity than other isoflavones, adult male mice showed significantly less exploration and higher levels of anxiety (Yu et al., 2013), thus confirming that perinatal exposure to estrogenic compounds can have long-term anxiogenic consequences in adulthood.

Then, we explored whether perinatal exposure to MXC could impact hippocampal dependent memory assessed through two complementary tests, namely the Y-maze spontaneous alternation test which evaluates spatial short-term memory and contextual fear conditioning which is dependent upon spatial and contextual cues and is a form of long-term hippocampus dependent memory. Indeed, it has been shown that a lesion of the hippocampus disrupts spatial memory performances in both the Y-maze spontaneous alternation test (Sanderson et al., 2009) and contextual fear conditioning (Anagnostaras et al., 2001).

For both tests, the obtained results showed a very significant effect of the treatment in both sexes: MXC-treated groups spent a greater proportion of time in the novel arm of the Y-maze and they also expressed a higher percentage of freezing time when re-exposed to the conditioning context. Although these results may appear surprising, a small body of literature has shown that EDCs may facilitate cognitive function in some cases. For example, exposure of male rats to 17α-ethinylestradiol during development results in enhanced spatial working memory during the acquisition phase of a Morris water maze test in male rats (Corrieri et al., 2007), a test that is also highly dependent upon hippocampus. In addition, adult male mice exposed to daidzein during the perinatal period, showed also a tendency to improve spatial learning and memory in adulthood (Yu et al., 2013). These results thus further suggest an organizational effect of estrogenic EDCs or phytoestrogens on hippocampus dependent memory.

In the Y-maze test, male mice exhibited higher performances compared to female mice. This finding is in accordance with previous studies showing that males generally outperform females on tasks that are considered to be hippocampus-dependent (for reviews see Galea et al., 2006; Luine, 2008). During the test phase of the conditioning to context task, opposite effect of sex on memory were observed. Indeed, control male mice exhibited less conditioned freezing to context than female. Both, the Y-maze and the contextual fear conditioning tasks are considered to be hippocampus dependent tasks. Nevertheless, both tasks assess different forms of hippocampus dependent memory and strongly differ in terms of emotional load. First, the Y-maze task is considered to assess short-term hippocampus dependent memory performances whereas the context test (in the fear conditioning to context task) assess long-term hippocampus dependent memory performances. Second, contextual fear conditioning is considered to assess hippocampus dependent memory when animals are exposed to emotionally laden stimuli which is not the case for the Y-maze task. In line with this, previous studies showed that temporary inactivation or permanent lesions of the amygdala, a brain region fully involved in emotional learning, severely interferes with contextual fear conditioning (Calandreau et al., 2005; Onishi and Xavier, 2010). On the contrary, in the Y-maze test (Sanderson et al., 2009), amygdala lesioned mice were still able to discriminate between a familiar and a novel arm. Consequently our findings suggest that male and female CD1 mice may have distinct memory performances depending on the type of memory targeted. These effects, although interesting, will deserve supplementary investigations to be fully understood.

The present enhancing effect of MXC on hippocampus dependent memory may be related to its well-established estrogenic action (Cummings, 1997). In line with this view, low doses of 17β-estradiol were previously reported to facilitate contextual fear conditioning in female rats (Barha et al., 2010). Moreover, aromatase-knockout (ArKO) male and female mice, which are unable to produce estrogens, exhibited deficits in spatial working memory assessed in the spontaneous alternation Y-maze test (Martin et al., 2003). These findings thus suggest that estrogenic EDCs could act during development to alter the organization of hippocampal dependent memory, even if we have not used a positive control (e.g., estradiol or the synthetic estrogen diethylstilbestrol). However, we cannot ascribe the observed behavioral effects only to an estrogenic action of MXC. In fact, it must be recognized that in addition to its documented estrogenicity, MXC may exert other effects on the developing brain; in fact, it has been shown to interact with multiple receptors (Ghosh et al., 1999), and to exert antiandrogenic action, as well (Gray et al., 1999).

Finally, Palanza et al. (2002) have shown a slight, but significant, perturbation of maternal behavior of females that had consumed very low doses of MXC during the last week of gestation. Even if the exposition time of MXC differs from our study (MXC was administered only during the gestational period, from day 11 to 17), we cannot exclude that variations in maternal care can have consequences for the subsequent behavioral development of the offspring and that some of the differences observed between MXC and control group may arise from such difference in maternal behavior.

Perinatal MXC treatment also led to a significant increase in cell survival in the DG of the hippocampus in both sexes compared to controls. Interestingly, the effect of MXC on hippocampal neurogenesis cannot be the consequence of confounding effects due to behavioral tests because independent groups of animals have been used to perform behavioral tests and to assess DG neurogenesis. Moreover, MXC treatment did not influence cell differentiation, because the proportion of surviving neurons, assessed through BrdU/NeuN double immunofluorescence labeling was stable in treated and untreated groups, suggesting that MXC affected only proliferation and/or survival processes. The present effect of MXC on hippocampal neurogenesis is very congruent with the enhancing effect of MXC on hippocampus dependent memory. Indeed, hippocampal adult neurogenesis has been shown to be a new form of plasticity that underlies hippocampus dependent learning and memory (Koehl and Abrous, 2011; Lafenetre et al., 2011). In particular, previous studies reported a positive correlation between reduced adult neurogenesis and deficits in contextual conditioning or spatial memory (Saxe et al., 2006; Winocur et al., 2006; Dupret et al., 2008; Imayoshi et al., 2008; Warner-Schmidt et al., 2008; Deng et al., 2009; Hernández-Rabaza et al., 2009; Snyder et al., 2009). Regarding our experimental conditions, it has been well demonstrated that contextual fear conditioning is dependent upon DG neurogenesis (Drew et al., 2010).

By contrast, data assessing the effect of estrogens treatment during the perinatal period on adult hippocampal neurogenesis are quite sparse. However, it is noticeable that 17β-estradiol treatment increases hippocampal cell proliferation in neonatal females and that this effect persisted until at least 3 weeks of age (Bowers et al., 2010). In addition, administering the aromatase inhibitor, formestane, or the estrogen receptor antagonist tamoxifen, during the perinatal period, significantly decreased the number of new cells in the DG observed 3 weeks later in males (Bowers et al., 2010). As a whole, these results suggest that the estrogenic properties of perinatal MXC may be responsible for both increased hippocampal memory and a higher rate of cell survival observed in the DG in adulthood.

It is interesting to notice that adult treatment with 17β-estradiol has been shown to increase both contextual fear conditioning (Barha et al., 2010) and adult DG neurogenesis in female rodents (see for example Ormerod et al., 2004; McClure et al., 2013), even if some variations in the effects may be observed according to doses, exposure durations or animal models. In addition, 17β-estradiol also led to a significant increase in the percentage of new neurons expressing the immediate early gene product zif268 in response to spatial memory retrieval (McClure et al., 2013), leading to the conclusion that these new neurons are activated during a spatial task, which may be functionally relevant (Veyrac et al., 2013).

Finally, it is striking to notice that the animals exposed to MXC and exhibiting therefore a higher state of anxiety, express also both a higher level of hippocampal neurogenesis and enhanced memory performances in the Y-maze and contextual fear conditioning tests. Although discussed, it is generally assumed that stressful situations and anxiety inhibit adult hippocampal neurogenesis and disrupt cognitive function. It has even been proposed that reduced neurogenesis was involved in the pathogenesis of anxiety related behavior (Snyder et al., 2011). Nevertheless, several experiments demonstrated that the link between anxiety or affective disorders and hippocampal neurogenesis is not trivial. Some experiments, using cranial irradiation or antimitotics agents report no effect of hippocampal neurogenesis ablation on anxiety related behaviors (Petrik et al., 2012 for review). Disruption of neurogenesis via inducible transgenic mouse models was even reported to increase or decrease anxiety behaviors (Revest et al., 2009; Onksen et al., 2011). Moreover, researches also provided evidence that some amount of stress or level of anxiety can be useful for learning and memory (Joels et al., 2006). Therefore, MXC treatment would have positive effects on hippocampal neurogenesis and hippocampus dependent memory through an increase in anxiety/stress level. Nevertheless, future studies will be required to fully understand the relationships between the present observed effects of MXC on anxiety, hippocampal neurogenesis and memory.

In conclusion, perinatal exposure to MXC induced durable effects on cognitive functions in adulthood such as increased anxiety and higher performances in hippocampal dependent memory tests, these later effects being correlated with a higher rate of cell survival in the DG of the hippocampus. Although these results correlate well with the known estrogenic properties of MXC, the exact mechanisms involved need to be determined. Moreover further investigations will be required to fully assess effect of MXC on different memory systems and other cognitive functions.

## Conflict of interest statement

The authors declare that the research was conducted in the absence of any commercial or financial relationships that could be construed as a potential conflict of interest.

## Footnotes

^1^http://ec.europa.eu/sanco_pesticides/public/index.cfm?event=activesubstance.selection&a=1

^2^http://www.epa.gov/oppsrrd1/REDs/methoxychlor_red.htm
